# Supplementary material for: Age and sex-specific risks of myocarditis and pericarditis following Covid-19 messenger RNA vaccines
Source: Nat Commun. 2022 Jun 25;13:3633. doi: 10.1038/s41467-022-31401-5 (PMC9233673; doi:10.1038/s41467-022-31401-5)
Supplement: Supplementary file 1 — Supplementary Information [file 41467_2022_31401_MOESM1_ESM.pdf]

Supplementary information

For the article “Age and sex-specific risks of myocarditis and pericarditis following Covid-19 messenger RNA Vaccines” by Le Vu et al.

Table S1. Number of mRNA vaccines doses administered 2

Table S2. Association between myocarditis and exposure to mRNA vaccines within 7 days, according to sex and age group (as illustrated in Figure 1)................................... 3

Table S3. Association between pericarditis and recent exposure to mRNA vaccines within 7 days, according to sex and age group (as illustrated in Figure 2). 4

Table S4. Sensitivity analyses (as illustrated in Figure S1). 5

Table S5. Number of doses needed for the occurrence of one case. 6

Figure S1. Sensitivity analyses. 7

Figure S2. Distribution of delay between vaccine receipt and hospitalization 8

Figure S3. Drug treatments up to 30 days post-discharge for myocarditis cases 9

Figure S4. Drug treatments up to 30 days post-discharge for pericarditis cases 10

Table S1. Number of mRNA vaccines doses administered

			BNT162b2		mRNA-1273		
Sex	Age	Population	dose 1	dose 2[a]	dose 1	dose 2[b]	Total doses
Female	12-17	2,496,548	1,699,996	1,459,109	82,405	61,359	3,302,869
	18-24	2,740,372	1,969,611	1,723,910	234,718	204,319	4,132,558
	25-29	1,862,776	1,246,795	1,118,612	161,156	143,668	2,670,231
	30-39	4,228,579	2,624,541	2,437,953	398,088	363,903	5,824,485
	40-50	4,817,922	2,931,425	2,870,105	498,891	480,392	6,780,813
Male	12-17	2,628,365	1,765,302	1,519,395	90,673	67,354	3,442,724
	18-24	2,870,504	1,993,604	1,733,962	262,168	222,008	4,211,742
	25-29	1,836,902	1,280,527	1,139,114	175,933	153,909	2,749,483
	30-39	4,005,898	2,657,436	2,427,920	415,457	372,146	5,872,959
	40-50	4,665,586	3,053,322	2,917,657	542,399	510,207	7,023,585
Overall		32,153,452	21,222,559	19,347,737	2,861,888	2,579,265	46,011,449

Study period from May 12, 2021 to October 31, 2021. [a] Among individuals that received the BNT162b2 vaccine as a second dose, 2.2% had received another vaccine for the first dose. [b] Among individuals receiving the mRNA-1273 vaccine as a second dose, 4.7% had received another vaccine for the first dose.

Table S2. Association between myocarditis and exposure to mRNA vaccines within 7 days, according to sex and age group (as illustrated in Figure 1).

			Male				Female			
Age	Exposure	Dose	Cases	Controls	OR (95% CI)[a]	aOR (95% CI)[b]	Cases	Controls	OR (95% CI)[a]	aOR (95% CI)[b]
12-17	Unexposed		92	1,119	Reference	Reference	22	248	Reference	Reference
	BNT162b2	1	3	49	0.71 (0.21-2.3)	0.86 (0.26-2.9)	1	2	6.1 (0.54-70)	8.5 (0.69-110)
		2	30	37	14 (7.4-26)	18 (9-35)	4	13	4.7 (1.1-20)	7.1 (1.5-33)
	mRNA-1273	1	0	1	-	-	0	0	-	-
		2	2	0	-	-	0	0	-	-
18-24	Unexposed		290	3,940	Reference	Reference	67	863	Reference	Reference
	BNT162b2	1	16	113	2.2 (1.3-3.8)	2.1 (1.2-3.7)	2	29	0.9 (0.2-4)	0.9 (0.2-4.1)
		2	88	130	12 (8.5-17)	13 (9.2-19)	12	25	7.8 (3.5-18)	9.6 (4.3-22)
	mRNA-1273	1	2	12	2.6 (0.56-13)	2.6 (0.56-13)	1	2	6.5 (0.57-76)	15 (1.1-200)
		2	38	12	46 (23-91)	44 (22-88)	11	4	33 (10-100)	41 (12-140)
25-29	Unexposed		138	1,743	Reference	Reference	30	348	Reference	Reference
	BNT162b2	1	8	32	3.3 (1.5-7.4)	4 (1.7-9.3)	0	12	-	-
		2	28	61	6.3 (3.8-10)	7.1 (4.2-12)	4	7	7.5 (1.8-31)	10 (2.1-47)
	mRNA-1273	1	1	5	2.4 (0.27-20)	3.4 (0.37-31)	0	2	-	-
		2	17	13	20 (9-45)	19 (8.3-43)	2	1	20 (1.8-220)	23 (2.1-270)
30-39	Unexposed		178	2,307	Reference	Reference	70	735	Reference	Reference
	BNT162b2	1	12	44	3.6 (1.9-7)	3.7 (1.9-7.4)	4	21	2 (0.66-5.9)	2.2 (0.64-7.4)
		2	25	72	4.8 (2.9-8)	5.7 (3.4-9.5)	5	19	2.7 (1-7.5)	4 (1.4-11)
	mRNA-1273	1	1	11	1.4 (0.17-11)	1.5 (0.19-12)	2	4	5.2 (0.94-28)	8.8 (1.5-52)
		2	24	7	41 (17-95)	45 (19-110)	1	4	2.6 (0.29-24)	1.4 (0.11-18)
40-50	Unexposed		132	1,471	Reference	Reference	59	568	Reference	Reference
	BNT162b2	1	4	55	0.75 (0.27-2.1)	0.94 (0.33-2.7)	1	13	0.7 (0.089-5.5)	0.88 (0.11-7.2)
		2	13	61	2.6 (1.4-5.1)	3 (1.5-5.9)	2	14	1.3 (0.29-6.1)	1.9 (0.39-9.3)
	mRNA-1273	1	2	9	2.4 (0.52-11)	2.4 (0.52-11)	0	2	-	-
		2	9	7	16 (5.6-45)	21 (7-64)	2	3	6.4 (1.1-39)	8 (1.1-57)
Overall	Unexposed		830	10,580	Reference	Reference	248	2,762	Reference	Reference
	BNT162b2	1	43	293	1.9 (1.4-2.7)	2 (1.4-2.8)	8	77	1.2 (0.55-2.5)	1.3 (0.57-2.8)
		2	184	361	7.6 (6.2-9.4)	8.7 (7-11)	27	78	4.3 (2.7-7)	5.9 (3.6-9.6)
	mRNA-1273	1	6	38	2.1 (0.88-5.1)	2.3 (0.96-5.6)	3	10	3.4 (0.92-12)	6.3 (1.6-25)
		2	90	39	32 (22-48)	34 (23-51)	16	12	14 (6.8-31)	17 (7.6-37)

[a] Odd-ratio (95% confidence interval) were obtained from univariable conditional logistic regression, adjusting for matching variables (sex, age and department of residence). [b] Odd-ratio (95% confidence interval) were obtained from multivariable conditional logistic regression, adjusting for matching variables (sex, age and department of residence), deprivation index, previous event in past 5 years of myocarditis, and history of SARS-CoV-2 infections in the month preceding the index date. Odds-ratio could not be calculated in categories where no case exposed to vaccine was recorded.

Table S3. Association between pericarditis and recent exposure to mRNA vaccines within 7 days, according to sex and age group (as illustrated in Figure 2).

			Male				Female			
Age	Exposure	Dose	Cases	Controls	OR (95% CI)[a]	aOR (95% CI)[b]	Cases	Controls	OR (95% CI)[a]	aOR (95% CI)[b]
12-17	Unexposed		52	545	Reference	Reference	28	286	Reference	Reference
	BNT162b2	1	0	11	-	-	0	18	-	-
		2	7	19	4.4 (1.7-12)	6.8 (2.3-20)	5	8	7.7 (2.1-28)	10 (2.5-41)
	mRNA-1273	1	0	0	-	-	0	1	-	-
		2	0	0	-	-	0	1	-	-
18-24	Unexposed		144	1,598	Reference	Reference	85	964	Reference	Reference
	BNT162b2	1	6	52	1.3 (0.55-3.2)	1.5 (0.6-3.5)	5	35	1.6 (0.61-4.4)	1.8 (0.65-4.8)
		2	21	42	6.3 (3.5-11)	6.3 (3.5-11)	13	32	4.7 (2.3-9.4)	5.9 (2.9-12)
	mRNA-1273	1	2	8	2.6 (0.5-13)	2.5 (0.5-13)	0	4	-	-
		2	8	9	11 (4.1-32)	11 (4.1-32)	0	3	-	-
25-29	Unexposed		82	904	Reference	Reference	70	757	Reference	Reference
	BNT162b2	1	4	33	1.4 (0.47-4.1)	1.4 (0.42-4.8)	3	28	1.2 (0.34-4)	1.5 (0.42-5.2)
		2	5	23	2.6 (0.94-6.9)	2.9 (1.1-8)	6	12	5.6 (2-15)	6.4 (2.3-18)
	mRNA-1273	1	1	6	1.9 (0.22-16)	1.9 (0.23-16)	1	5	2.4 (0.27-21)	2.4 (0.27-21)
		2	2	4	5.6 (1-31)	7.5 (1.2-45)	0	4	-	-
30-39	Unexposed		215	2,364	Reference	Reference	147	1,517	Reference	Reference
	BNT162b2	1	8	63	1.5 (0.68-3.1)	1.4 (0.65-3.2)	5	27	2 (0.74-5.5)	3.1 (1.1-8.5)
		2	13	64	2.5 (1.3-4.7)	2.4 (1.2-4.6)	9	44	2.2 (1-4.6)	2 (0.9-4.6)
	mRNA-1273	1	2	21	1.1 (0.25-4.7)	1.1 (0.25-4.7)	1	9	1.1 (0.15-9.1)	1.2 (0.15-9.5)
		2	3	7	5 (1.3-19)	4.9 (1.3-19)	5	5	12 (3.2-45)	20 (3.5-110)
40-50	Unexposed		286	2,827	Reference	Reference	160	1,636	Reference	Reference
	BNT162b2	1	7	86	0.79 (0.36-1.8)	0.85 (0.36-2)	5	45	1.1 (0.43-2.9)	1.5 (0.56-3.9)
		2	7	90	0.79 (0.36-1.7)	0.88 (0.4-1.9)	7	40	1.8 (0.79-4)	1.5 (0.59-4)
	mRNA-1273	1	1	16	0.6 (0.079-4.6)	0.62 (0.08-4.7)	0	8	-	-
		2	3	17	1.7 (0.51-5.9)	1.4 (0.32-5.9)	5	4	12 (3.3-46)	13 (3.5-49)
Overall	Unexposed		779	8,238	Reference	Reference	490	5,160	Reference	Reference
	BNT162b2	1	25	245	1.1 (0.71-1.7)	1.2 (0.74-1.8)	18	153	1.2 (0.74-2)	1.6 (0.93-2.6)
		2	53	238	2.5 (1.8-3.4)	2.7 (1.9-3.7)	40	136	3.1 (2.2-4.5)	3.4 (2.3-5.1)
	mRNA-1273	1	6	51	1.2 (0.53-2.9)	1.3 (0.54-3)	2	27	0.77 (0.18-3.3)	0.93 (0.22-4)
		2	16	37	4.8 (2.6-8.6)	5 (2.7-9.2)	10	17	6.2 (2.8-14)	6.7 (2.9-16)

[a] Odd-ratio (95% confidence interval) were obtained from univariable conditional logistic regression, adjusting for matching variables (sex, age and department of residence). [b] Odd-ratio (95% confidence interval) were obtained from multivariable conditional logistic regression, adjusting for matching variables (sex, age and department of residence), deprivation index, previous event in past 5 years of pericarditis, and history of SARS-CoV-2 infections in the month preceding the index date. Odds-ratio could not be calculated in categories where no case exposed to vaccine was recorded.

Table S4. Sensitivity analyses (as illustrated in Figure S1).

			Myocarditis				Pericarditis			
Model	Exposition	Dose	Cases	Controls	OR (95% CI)	aOR (95% CI)	Cases	Controls	OR (95% CI)	aOR (95% CI)
A	Unexposed		1078	13342	Reference	Reference	1269	13398	Reference	Reference
	BNT162b2	1	51	370	1.7 (1.3-2.4)	1.8 (1.3-2.5)	43	398	1.1 (0.83-1.6)	1.3 (0.92-1.8)
		2	211	439	6.9 (5.7-8.4)	8.1 (6.7-9.9)	93	374	2.7 (2.2-3.5)	2.9 (2.3-3.8)
	mRNA-1273	1	9	48	2.4 (1.2-5)	3 (1.4-6.2)	8	78	1.1 (0.52-2.2)	1.2 (0.56-2.4)
		2	106	51	27 (19-39)	30 (21-43)	26	54	5.3 (3.3-8.4)	5.5 (3.3-9)
B	Unexposed		460	5115	Reference	Reference	512	5128	Reference	Reference
	BNT162b2	1	29	180	1.8 (1.2-2.6)	1.9 (1.2-2.9)	16	213	0.74 (0.44-1.2)	0.79 (0.45-1.4)
		2	34	130	3.1 (2.1-4.6)	3.7 (2.4-5.6)	23	116	2.1 (1.3-3.3)	2.1 (1.3-3.5)
	mRNA-1273	1	4	20	2.2 (0.75-6.5)	3.1 (1-9.4)	5	36	1.4 (0.53-3.5)	1.6 (0.61-4.1)
		2	31	24	16 (9.1-28)	18 (10-32)	10	21	5 (2.3-11)	4 (1.8-9.3)
C	Unexposed		1020	13246	Reference	Reference	1230	13301	Reference	Reference
	BNT162b2	1	50	370	1.8 (1.4-2.5)	1.9 (1.4-2.6)	43	398	1.2 (0.85-1.6)	1.3 (0.93-1.8)
		2	211	438	7.4 (6.1-9)	8.2 (6.7-10)	93	372	2.8 (2.2-3.6)	3 (2.3-3.8)
	mRNA-1273	1	9	48	2.6 (1.2-5.3)	3 (1.4-6.2)	8	78	1.1 (0.53-2.3)	1.2 (0.56-2.4)
		2	105	51	28 (20-40)	28 (19-40)	26	53	5.4 (3.3-8.6)	5.6 (3.4-9.3)
D	Unexposed		1022	13342	Reference	Reference	1247	13398	Reference	Reference
	BNT162b2	1	48	370	1.7 (1.2-2.3)	1.8 (1.3-2.5)	43	398	1.2 (0.85-1.6)	1.3 (0.96-1.9)
		2	196	439	6.8 (5.6-8.3)	8 (6.5-9.8)	88	374	2.6 (2.1-3.4)	2.8 (2.2-3.6)
	mRNA-1273	1	9	48	2.5 (1.2-5.2)	3.1 (1.5-6.5)	7	78	0.96 (0.44-2.1)	1 (0.48-2.3)
		2	100	51	26 (18-37)	28 (19-40)	23	54	4.8 (2.9-7.8)	4.9 (2.9-8.4)
E	Unexposed		974	13335	Reference	Reference	1120	13390	Reference	Reference
	BNT162b2	1	47	370	1.8 (1.3-2.4)	1.8 (1.3-2.5)	40	398	1.2 (0.89-1.8)	1.3 (0.92-1.8)
		2	208	439	7.5 (6.2-9.2)	8 (6.6-9.8)	88	374	2.9 (2.3-3.7)	2.9 (2.3-3.8)
	mRNA-1273	1	9	48	2.8 (1.3-5.8)	3 (1.5-6.3)	8	78	1.1 (0.55-2.4)	1.2 (0.56-2.4)
		2	101	51	28 (19-39)	30 (21-43)	24	54	5.6 (3.4-9.1)	5.5 (3.4-9.1)

Legend: Univariable and multivariable odds ratios of contracting myocarditis or pericarditis within 7 days of vaccination with each mRNA vaccine obtained in the main analysis (model A), and in the following sensitivity analyses: restricted to the period prior to the modification in vaccine product information (i.e. July 19, 2021), warning against myocarditis and pericarditis as adverse events (model B); excluding patients with history of SARS-CoV-2 infection in past month (model C); excluding patients with both conditions (model D); excluding patients with history of the condition of interest within 5 years (model E).

Table S5. Number of doses needed for the occurrence of one case.

				Myocarditis	Pericarditis
Sex	Age	Vaccine	Dose	N (95% CI)[a]	N (95% CI)[a]
Male	12-17	BNT162b2	1	-	-
			2	52,300 (38,200;74,100)	248,300 (113,600;587,800)
		mRNA-1273	1	-	-
			2	-	-
	18-24	BNT162b2	1	240,700 (91,300;653,400)	-
			2	21,100 (17,400;26,000)	97,200 (60,600;160,500)
		mRNA-1273	1	-	-
			2	5,900 (4,400;8,000)	29,900 (14,500;63,200)
	25-29	BNT162b2	1	211,600 (86,600;537,300)	-
			2	47,000 (31,800;71,400)	344,100 (95,100;1,348,000)
		mRNA-1273	1	-	-
			2	9,400 (5,900;15,200)	87,100 (18,800;430,300)
	30-39	BNT162b2	1	301,700 (141,800;661,500)	-
			2	116,900 (74,900;187,100)	320,100 (124,100;854,300)
		mRNA-1273	1	-	-
			2	15,600 (10,700;23,000)	152,900 (37,900;633,300)
	40-50	BNT162b2	1	-	-
			2	335,600 (151,000;780,300)	-
		mRNA-1273	1	-	-
			2	58,600 (30,800;115,100)	-
Female	12-17	BNT162b2	1	-	-
			2	414,500 (158,400;1,303,000)	316,300 (138,500;825,300)
		mRNA-1273	1	-	-
			2	-	-
	18-24	BNT162b2	1	-	-
			2	159,000 (90,800;294,400)	158,100 (86,800;302,400)
		mRNA-1273	1	249,300 (33,200;2,033,000)	-
			2	18,700 (11,000;33,400)	-
	25-29	BNT162b2	1	-	-
			2	307,400 (118,600;910,500)	218,700 (90,400;564,100)
		mRNA-1273	1	-	-
			2	73,600 (20,100;309,400)	-
	30-39	BNT162b2	1	-	775,100 (214,700;2,924,000)
			2	646,000 (213,300;2,112,000)	-
		mRNA-1273	1	222,200 (50,000;1,061,000)	-
			2	-	75,400 (31,000;188,200)
	40-50	BNT162b2	1	-	-
			2	-	-
		mRNA-1273	1	-	-
			2	270,500 (60,200;1,324,000)	102,500 (41,000;263,300)

[a] Numbers are only calculated in case of a positive association.

Figure S1. Sensitivity analyses.

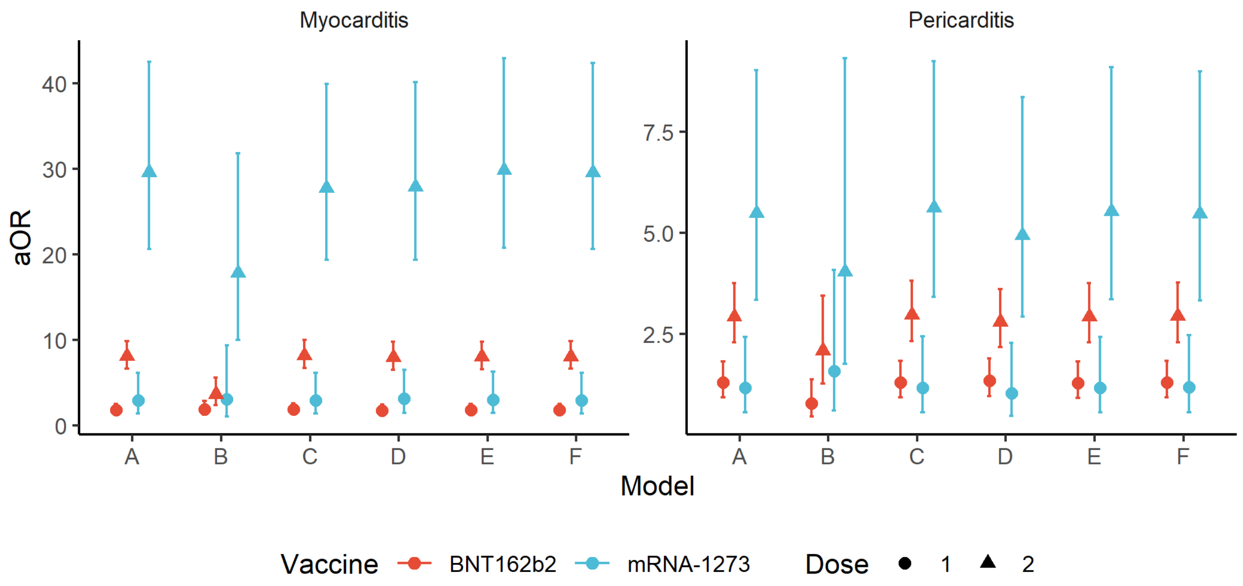

Legend: Adjusted odds ratios of contracting myocarditis or pericarditis within 7 days of vaccination with each mRNA vaccine obtained in the main analysis (model A), and in the following sensitivity analyses: restricted to the period prior to the modification in vaccine product information (i.e. July 19, 2021), warning against myocarditis and pericarditis as adverse events (model B); excluding patients with history of SARS-CoV-2 infection in past month (model C); excluding patients with both conditions (model D); excluding patients with history of the condition of interest within 5 years (model E); excluding control persons with a hospitalization within a month prior to the index date (model F). Colors denote the type of vaccine and the shape of point estimate denotes the ranking of dose vaccine. Centre value are aOR point estimates and error bars represent 95% confidence intervals. Total number of cases for models A through F were respectively 1612, 606, 1548, 1525, 1486 and 1612 for myocarditis analysis, and 1613, 622, 1571, 1576, 1440 and 1613 for pericarditis analysis.

Figure S2. Distribution of delay between vaccine receipt and hospitalization

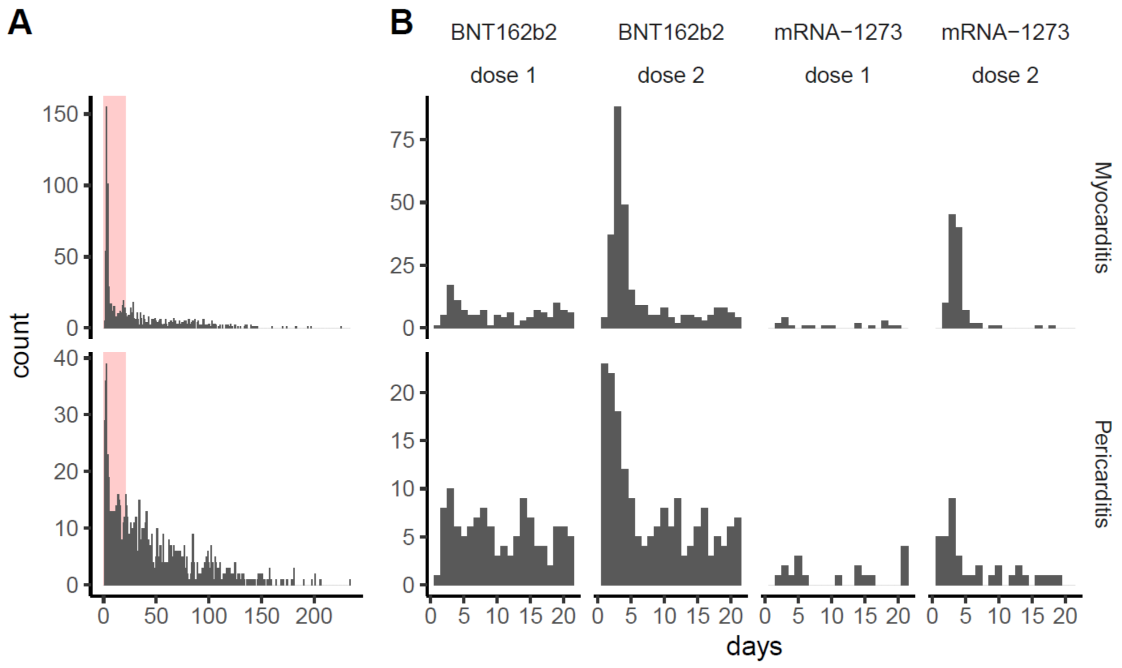

Legend: A panel shows delays for all cases in days, by condition. B panel shows delays restricted to the 21 days of exposure post vaccination (red portion of panel A), by vaccine and dose.

Figure S3. Drug treatments up to 30 days post-discharge for myocarditis cases

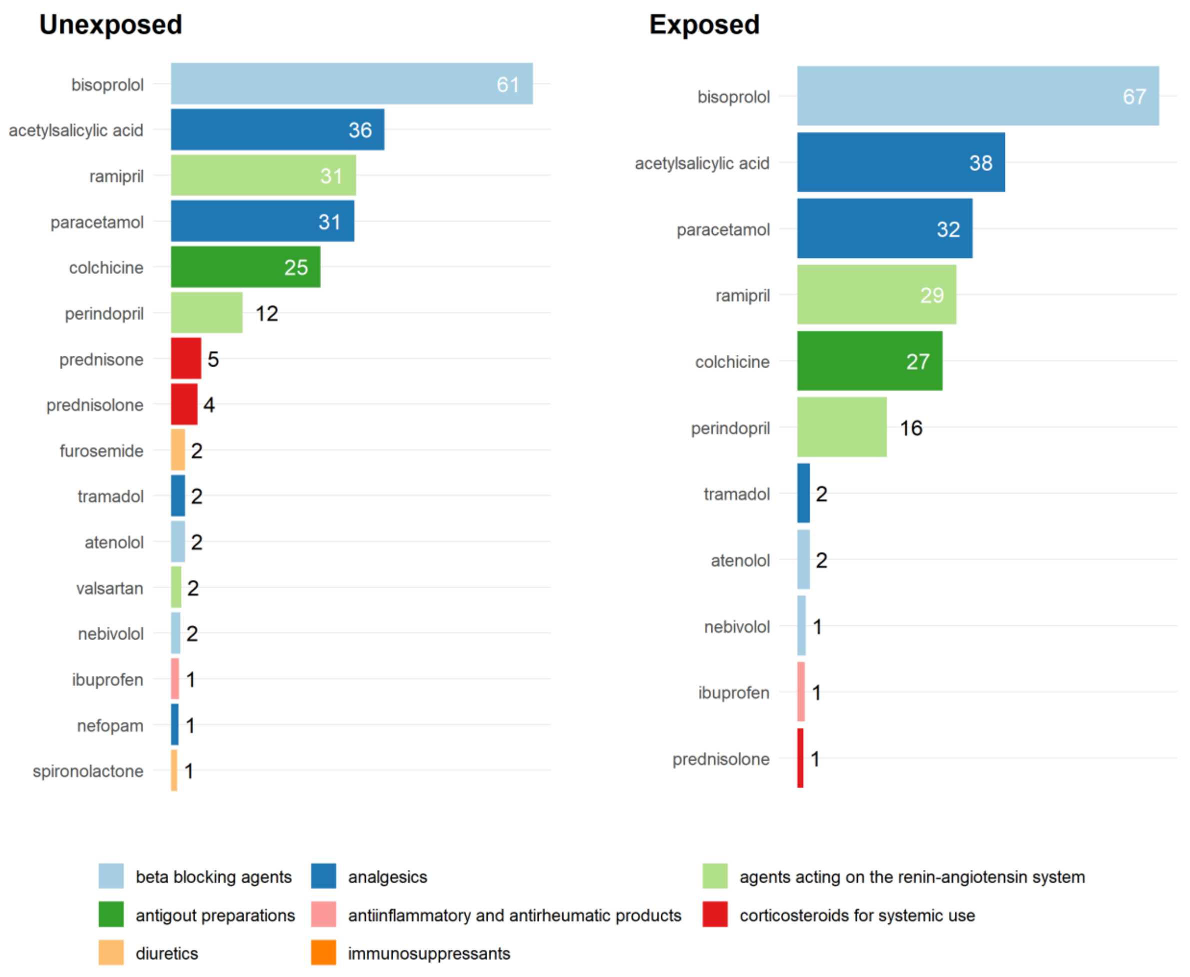

Legend: Proportion of patients receiving an agent up to 30 days post hospital discharge are represented by descending order of frequency, and colored by therapeutic class relevant to the treatment of myocarditis. Exposed refers to patients with myocarditis acquired within 21 days of vaccination.

Figure S4. Drug treatments up to 30 days post-discharge for pericarditis cases

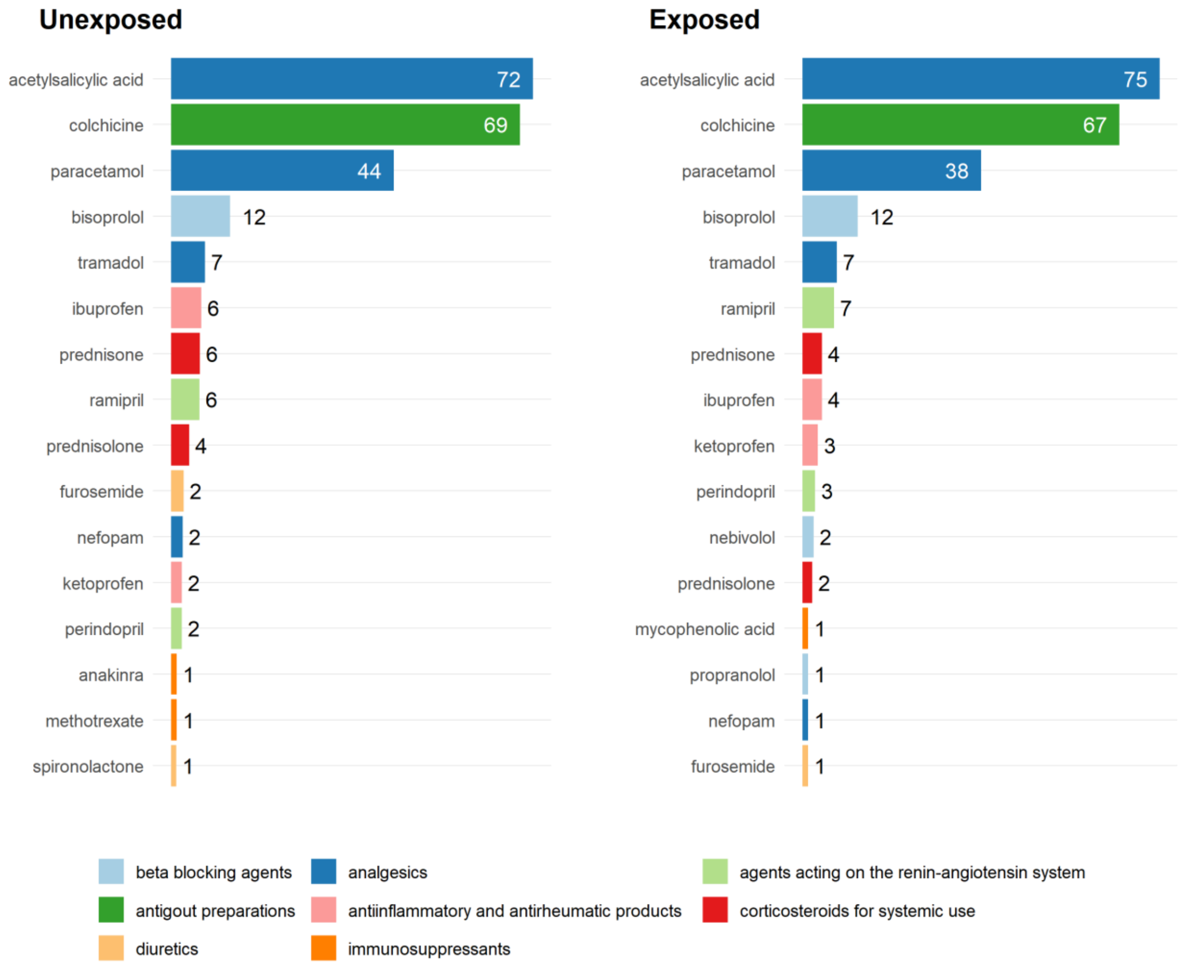

Legend: Proportion of patients receiving an agent up to 30 days post hospital discharge are represented by descending order of frequency and colored by therapeutic class relevant to the treatment of pericarditis. Exposed refers to patients with pericarditis acquired within 21 days of vaccination.
