# Supplementary material for: Age and sex-specific risks of myocarditis and pericarditis following Covid-19 messenger RNA vaccines
Source: Nat Commun. 2022 Jun 25;13:3633. doi: 10.1038/s41467-022-31401-5 (PMC9233673; doi:10.1038/s41467-022-31401-5)
Supplement: Supplementary file 3 — Reporting Summary [file 41467_2022_31401_MOESM3_ESM.pdf]

nature portfolio

Corresponding author(s): Stéphane Le Vu

Last updated by author(s): May 25, 2022

Reporting Summary

Nature Portfolio wishes to improve the reproducibility of the work that we publish. This form provides structure for consistency and transparency in reporting. For further information on Nature Portfolio policies, see our Editorial Policies and the Editorial Policy Checklist.

Statistics

For all statistical analyses, confirm that the following items are present in the figure legend, table legend, main text, or Methods section.

n/a	Confirmed	
☐	☒	The exact sample size (*n*) for each experimental group/condition, given as a discrete number and unit of measurement
☐	☒	A statement on whether measurements were taken from distinct samples or whether the same sample was measured repeatedly
☒	☐	The statistical test(s) used AND whether they are one- or two-sided<br>*Only common tests should be described solely by name; describe more complex techniques in the Methods section.*
☐	☒	A description of all covariates tested
☐	☒	A description of any assumptions or corrections, such as tests of normality and adjustment for multiple comparisons
☐	☒	A full description of the statistical parameters including central tendency (e.g. means) or other basic estimates (e.g. regression coefficient) AND variation (e.g. standard deviation) or associated estimates of uncertainty (e.g. confidence intervals)
☒	☐	For null hypothesis testing, the test statistic (e.g. *F*, *t*, *r*) with confidence intervals, effect sizes, degrees of freedom and *P* value noted<br>*Give P values as exact values whenever suitable.*
☒	☐	For Bayesian analysis, information on the choice of priors and Markov chain Monte Carlo settings
☒	☐	For hierarchical and complex designs, identification of the appropriate level for tests and full reporting of outcomes
☒	☐	Estimates of effect sizes (e.g. Cohen's *d*, Pearson's *r*), indicating how they were calculated

Our web collection on statistics for biologists contains articles on many of the points above.

Software and code

Policy information about availability of computer code

Data collection	SAS Enterprise Guide version 4.3 software (SAS Institute, Cary, North Carolina)
Data analysis	R software version 4.1.3, and survival package version 3.2-13. Reproducible code is publicly available at https://doi.org/10.5281/zenodo.6583550

For manuscripts utilizing custom algorithms or software that are central to the research but not yet described in published literature, software must be made available to editors and reviewers. We strongly encourage code deposition in a community repository (e.g. GitHub). See the Nature Portfolio guidelines for submitting code & software for further information.

Data

Policy information about availability of data

All manuscripts must include a data availability statement. This statement should provide the following information, where applicable:

- Accession codes, unique identifiers, or web links for publicly available datasets
- A description of any restrictions on data availability
- For clinical datasets or third party data, please ensure that the statement adheres to our policy

According to data protection and the French regulation, the authors cannot publicly release the data from the French national health data system (SNDS). However, any person or structure, public or private, for-profit or nonprofit, is able to access SNDS data upon authorization from the French Data Protection Office (CNIL Commission Nationale de l'Informatique et des Libertés) to carry out a study, a research, or an evaluation of public interest (https://www.snds.gouv.fr/SNDS/Processus-d-acces-aux-donnees and https://www.indsante.fr/).

Field-specific reporting

Please select the one below that is the best fit for your research. If you are not sure, read the appropriate sections before making your selection.

☒ Life sciences ☐ Behavioural & social sciences ☐ Ecological, evolutionary & environmental sciences

For a reference copy of the document with all sections, see nature.com/documents/nr-reporting-summary-flat.pdf

Life sciences study design

All studies must disclose on these points even when the disclosure is negative.

Sample size	We included all cases of the outcomes of interest in the study period, and 10 control subjects per each. A posthoc power calculation indicates that we are able to detect with a power of 80% an OR of 1.5, with 1000 cases (we had ~1600), 10 controls each, and with the observed level of population exposure.
Data exclusions	We excluded persons under age 12 -- not eligible for vaccination at the time, and over 50, for whom the outcomes of interest are less common, and have more chance to be confused with other cardiovascular diseases.
Replication	Results could not be reproduced. In this observational study, we analyzed all cases occurring in the country during a period.
Randomization	Randomization did not intervene in group allocation. Control subjects were randomized from the unaffected population and matched with cases by age, gender and region .
Blinding	Blinding is not relevant in this observational study performed on administrative electronic records.

Reporting for specific materials, systems and methods

We require information from authors about some types of materials, experimental systems and methods used in many studies. Here, indicate whether each material, system or method listed is relevant to your study. If you are not sure if a list item applies to your research, read the appropriate section before selecting a response.

Materials & experimental systems

n/a	Involved in the study	
☒	☐	Antibodies
☒	☐	Eukaryotic cell lines
☒	☐	Palaeontology and archaeology
☒	☐	Animals and other organisms
☐	☒	Human research participants
☒	☐	Clinical data
☒	☐	Dual use research of concern

Methods

n/a	Involved in the study	
☒	☐	ChIP-seq
☒	☐	Flow cytometry
☒	☐	MRI-based neuroimaging

Human research participants

Policy information about studies involving human research participants

Population characteristics	3225 patients aged 12-50 (median 30 y, 30% female, 58% vaccinated) admitted to hospital with carditis from 12 May 2021 to 31 october 2021 and 32,250 control subjects (median 30 y, 30% female, 50% vaccinated) from general population.
Recruitment	Individual data were obtained from the National Health Data System (SNDS), gathering the French hospital discharge database (PMSI) and nationwide databases for Covid-19 vaccination (VAC-SI) and testing (SI-DEP). Data collection is systematic and there is no potential self selection.
Ethics oversight	Our research group (EPI-PHARE) has a regulatory permanent access to the data from the SNDS. This permanent access is given according the French Decree No. 2016-1871 of December 26, 2016 relating to the processing of personal data called "National Health Data System" and French law articles Art. R. 1461-13 and 14. This study was declared prior to initiation on the EPI-PHARE registry of studies requiring the use of the SNDS (n° EP-0311).

Note that full information on the approval of the study protocol must also be provided in the manuscript.
